# Validation of the Brief Pathological Narcissism Inventory and Its Association with Cyber Intimate Partner Violence in Portuguese Emerging Adults

**DOI:** 10.3390/ejihpe16100155

**Published:** 2026-10-01

**Authors:** Mónica Rego, Sónia Caridade

**Affiliations:** Psychology Research Centre, School of Psychology, University of Minho, Campus de Gualtar, 4710-057 Braga, Portugal; monicasrego@gmail.com

**Keywords:** cyber intimate partner violence, pathological narcissism, vulnerable narcissism, grandiose narcissism, perpetration, emerging adults

## Abstract

Background: Cyber intimate partner violence (C-IPV) refers to abusive and controlling behaviors enacted through digital technologies toward a current or former intimate partner. Previous research suggests that pathological narcissism may be associated with C-IPV. Methods: This study examined the psychometric properties of the Brief Pathological Narcissism Inventory (B-PNI), the frequency of self-reported C-IPV perpetration and related behaviors, and the association between C-IPV perpetration and pathological narcissism among emerging adults. The sample comprised 371 participants aged 18–25 years (M = 21.52, SD = 1.97), of whom 78.7% were female. Results: Confirmatory factor analysis provided support for the a priori two-factor model of the B-PNI (CFI = 0.982, TLI = 0.965, RMSEA = 0.076), comprising grandiose and vulnerable narcissism. The B-PNI showed satisfactory internal consistency and theoretically expected associations with psychopathological symptoms and broader personality dimensions. Overall, 79.5% of participants reported at least one C-IPV behavior, with 61.5% reporting online control, 62.0% online jealousy, and 31.5% online intrusive behaviors. C-IPV perpetration was positively and significantly associated with both grandiose and vulnerable narcissism, although the associations were small in magnitude. Conclusions: The findings provide preliminary support for the use of the B-PNI among Portuguese emerging adults and indicate that pathological narcissism is associated with C-IPV perpetration. Given the modest magnitude of these associations, narcissistic traits should be considered as one of multiple individual and relational factors potentially involved in C-IPV.

## 1. Introduction

Pathological narcissism is a multidimensional construct characterized by maladaptive self-regulatory processes, emotional vulnerability, and interpersonal dysfunction (Pincus et al., 2009). Contemporary conceptualizations generally distinguish between two major dimensions: grandiose and vulnerable narcissism (Pincus & Lukowitsky, 2010).

Grandiose narcissism is characterized by dominance, entitlement, exhibitionism, interpersonal exploitation, and an excessive need for admiration, whereas vulnerable narcissism is associated with insecurity, hypersensitivity to criticism, emotional instability, shame, and fear of rejection (Miller et al., 2011).

Although grandiose and vulnerable narcissism share core features of narcissistic functioning, they show distinct patterns of association with psychological adjustment and broader personality characteristics. Vulnerable narcissism has been particularly associated with psychological distress, including anxiety and depressive symptoms (Brailovskaia et al., 2020, 2021), whereas evidence regarding grandiose narcissism and psychological symptoms appears less consistent (Brailovskaia et al., 2020). Pathological narcissism has also been linked to somatic distress, although evidence suggests that this relationship may operate indirectly through heightened sensitivity to bodily sensations (Kealy et al., 2018). Furthermore, grandiose and vulnerable narcissism exhibit distinct personality profiles within the Big Five framework, particularly regarding extraversion and neuroticism, while sharing an antagonistic component reflected in lower agreeableness (Zajenkowski & Szymaniak, 2021). These differential patterns of association with psychopathological symptoms and broader personality dimensions provide a theoretical basis for examining the construct validity of the B-PNI through its associations with theoretically related constructs.

Several instruments assessing narcissistic traits have been validated for the Portuguese population, including the Narcissistic Personality Inventory (NPI-13) and the Hypersensitive Narcissism Scale (HSNS) (Pechorro et al., 2016, 2018, 2019; Pereira & Paixão, 2019). However, these measures primarily assess either grandiose or hypersensitive narcissistic features separately. The Brief Pathological Narcissism Inventory (B-PNI; Schoenleber et al., 2015) provides a more comprehensive assessment by simultaneously capturing grandiose and vulnerable dimensions of pathological narcissism. The B-PNI is a reduced version of the Pathological Narcissism Inventory (PNI; Pincus et al., 2009) and has demonstrated adequate psychometric properties across different cultural contexts (Diguer et al., 2020; Fossati et al., 2018; Malaeb et al., 2023). However, to date, the B-PNI has not been validated for Portuguese emerging adults.

### 1.1. Pathological Narcissism and Cyber Intimate Partner Violence

Beyond its associations with personality and psychological adjustment, pathological narcissism may be particularly relevant to understanding maladaptive and aggressive behaviors within intimate relationships. Characteristics associated with narcissistic functioning, including entitlement, interpersonal exploitation, sensitivity to rejection, unstable self-esteem, and difficulties in emotion regulation, may contribute to controlling or aggressive responses when individuals experience perceived rejection, humiliation, or threats to their self-image.

This relationship may be especially relevant during emerging adulthood, a developmental period characterized by increasing exploration of intimacy and the development of more enduring and meaningful romantic relationships (Arnett, 2000; Boisvert et al., 2023). Information and Communication Technologies (ICTs) have also become central to the establishment and maintenance of intimate relationships among young people and emerging adults, enabling frequent and sometimes continuous interaction through social networking sites, messaging applications, video calls, and other digital platforms (Burke et al., 2011; Caridade et al., 2020). Together, these developmental and relational characteristics make emerging adults a particularly relevant population in which to examine personality-related factors associated with C-IPV. Despite their relational benefits, digital technologies may also facilitate abusive interpersonal dynamics, including cyber intimate partner violence (C-IPV).

The literature has used different terms to describe this phenomenon, including Cyber Dating Abuse, Digital Dating Abuse, and Cyber Intimate Partner Violence (Borrajo et al., 2015; Caridade et al., 2019; Reed et al., 2016). In the present study, C-IPV refers to abusive and controlling behaviors perpetrated through digital technologies toward a current or former intimate partner. Such behaviors may include threats, humiliation, harassment, monitoring, invasion of privacy, and coercive control enacted through digital means (Borrajo et al., 2015; Gámez-Guadix et al., 2018; Villora et al., 2019). Research has commonly distinguished between direct aggression, such as insults, threats, and public humiliation, and controlling behaviors, such as monitoring online activity, excessive messaging, and checking social media interactions (Borrajo et al., 2015; Caridade & Braga, 2019).

Digital environments may provide particular opportunities for these behaviors by enabling continuous access to intimate partners, reducing temporal and physical barriers, and facilitating surveillance and monitoring (Bhogal & Wallace, 2022; Peterson & Densley, 2017). Online abuse also frequently co-occurs with offline intimate partner violence, suggesting continuity between digital and face-to-face abusive relational dynamics (Caridade et al., 2020; Cava et al., 2020).

Studies have documented substantial frequencies of C-IPV behaviors among adolescents and emerging adults, although estimates vary considerably according to the terminology, instruments, samples, and operational definitions employed (Caridade et al., 2019). Portuguese studies have similarly identified substantial proportions of young people reporting C-IPV behaviors, particularly those involving online control (Caridade & Braga, 2019; Caridade et al., 2020; Monteiro et al., 2023). Importantly, controlling and jealousy-related digital behaviors may sometimes be normalized or interpreted as expressions of relational involvement, potentially obscuring their abusive nature.

Regarding gender, recent meta-analytic evidence suggests that overall rates of C-IPV perpetration and victimization do not differ significantly between males and females, although gender-related patterns may vary according to developmental factors, with age and educational level emerging as significant moderators of differences in victimization (Erbiçer et al., 2025).

The perpetration of C-IPV has been associated with several psychosocial, relational, and personality-related factors, including jealousy, emotional dysregulation, insecure attachment, sexist beliefs, and maladaptive personality traits (Peskin et al., 2017). The General Aggression Model (GAM; Anderson & Bushman, 2002) provides a useful framework for understanding these associations, proposing that aggressive behavior results from interactions between personal and situational inputs that influence cognitive, affective, and physiological states and, ultimately, behavioral responses. Within this framework, personality characteristics constitute relevant personal inputs that may increase the likelihood of aggressive responses under particular interpersonal conditions (Bui & Pasalich, 2021; Warburton & Anderson, 2018).

Pathological narcissism may constitute one such personality-related factor. Digital environments may be particularly salient for individuals with narcissistic traits because online interactions provide opportunities for self-presentation, social validation, and interpersonal monitoring (Buffardi & Campbell, 2008). Features associated with pathological narcissism, including entitlement, jealousy, rejection sensitivity, interpersonal control, and threats to self-esteem, may therefore contribute to abusive or controlling behaviors toward intimate partners in digital contexts.

Several studies have reported associations between narcissistic traits and C-IPV perpetration (Branson & March, 2021; Lambe et al., 2018; March et al., 2021; Zerach, 2016). Vulnerable narcissism may be particularly relevant because heightened sensitivity to rejection and fears of abandonment may contribute to controlling and monitoring behaviors toward intimate partners (Branson & March, 2021; March et al., 2021). However, findings remain inconsistent, with other studies identifying associations involving grandiose narcissism or failing to identify significant relationships between particular narcissistic traits and cyber aggression (Bhogal & Wallace, 2022; Pabian et al., 2015). These mixed findings reinforce the need to examine grandiose and vulnerable narcissism separately.

Despite growing international interest in this relationship, evidence concerning pathological narcissism and C-IPV among Portuguese emerging adults remains limited. Examining this association alongside the psychometric evaluation of the B-PNI may therefore contribute both to the assessment of pathological narcissism in the Portuguese context and to a better understanding of personality-related factors associated with digitally mediated intimate partner violence.

### 1.2. Present Study

The present study had three objectives: (i) to translate and validate the Brief Pathological Narcissism Inventory (B-PNI) for Portuguese emerging adults by examining its factorial structure, reliability, and construct validity, including associations with broader personality dimensions and psychopathological symptoms; (ii) to examine the frequency of self-reported C-IPV perpetration and related behaviors within the study sample; and (iii) to examine the associations between C-IPV perpetration and pathological narcissism, considering both grandiose and vulnerable dimensions.

## 2. Materials and Methods

### 2.1. Participants

The study targeted emerging adults aged 18–25 years. Although conceptualizations of emerging adulthood may encompass a broader age range, the 18–25-year range was defined a priori in the study protocol and applied throughout participant recruitment and data collection. Eligibility criteria included being aged between 18 and 25 years and being currently involved in, or having previously been involved in, a romantic relationship. Participants who did not meet the predefined age criterion or who reported never having been in a romantic relationship were excluded from the analyses.

The initial sample consisted of 671 participants, of whom 300 were excluded after applying these eligibility criteria. The final sample consisted of a total of 371 young Portuguese (292 females and 79 males), aged between 18 and 25 years (M = 21.52, SD = 1.973). 94.1% of the participants had Portuguese nationality, 47.2% had completed secondary school, 44.2% had a university degree, while the remaining participants had different levels of education. Regarding romantic relationships, 67.7% of the participants were in a romantic relationship at the time of completing the questionnaire, while 32.3% were not in a romantic relationship at the time of completing the questionnaire, but reported having had romantic relationships in the past. The mean duration of the relationship was 27.44 months, with a standard deviation of 24.627. The majority of the sample (45.6%) reported being in a relationship for more than 3 years, 30.5% reported being in a relationship for less than 1 year and 24% reported being in a relationship between 1 and 3 years.

### 2.2. Instruments

The socio-demographic questionnaire included open-ended questions about age and occupation, and closed-ended questions about gender, sexual orientation, nationality, educational attainment, marital status, occupational situation, socioeconomic level, and whether they had ever been in a romantic relationship.

The Cyber Dating Questionnaire—Q_A (Sánchez-Jiménez et al., 2015), validated for the Portuguese population by Caridade et al. (2025). The Cyberdating Q_A is designed to assess adolescents’ use of digital technologies within intimate relationships and to capture different forms of CDA. It consists of six subscales assessing different positive and negative dimensions of adolescent online dating relationships. In this study only the three negative subscales specifically assessing different forms of online aggression were used. The instrument comprised 13 items rated on a five-point Likert scale ranging from 0 (never) to 4 (always): 1. Online Jealousy, assessed through four items (e.g., “I feel jealous when my partner posts provocative photos on their social media profile”); 2. Online Intrusiveness, measured through four items (e.g., “When we argue and my partner does not reply to me, I leave them many private messages”); and 3. Online Control, assessed through five items (e.g., “I have added my partner’s friends to monitor them”). In the original validation study, all dimensions demonstrated good internal consistency, with Cronbach’s alpha coefficients ranging from 0.79 to 0.85 (Sánchez-Jiménez et al., 2015). In the present study, Cronbach’s alpha coefficients across the three C-IPV dimensions ranged from 0.46 to 0.76. Given the low internal consistency observed for some dimensions, findings involving these subscales should be interpreted with caution.

The Brief Symptom Inventory (BSI) was validated by Canavarro (1999) for the Portuguese population. It assesses psychopathological symptoms and consists of 53 items on a four-point Likert scale: 0. “Never”; 1. “A few times”; 2. “Sometimes”; 3. “Often”; and 4. “Very often”. These items can be divided into nine symptom dimensions, such as somatization, obsessions and compulsions, interpersonal sensitivity, depression, anxiety, hostility, phobic anxiety, paranoid ideation, and psychoticism. In the present study, only 19 questions were presented on the Somatization, Depression, and Anxiety dimensions, which have a Cronbach’s alpha of α = 0.80, α = 0.73, and α = 0.77, respectively, so this instrument has adequate internal consistency (Canavarro, 1999). In this study, the BSI had Cronbach’s alphas of α = 0.91, α = 0.92, and α = 0.89 for the somatization, depression, and anxiety dimensions, respectively.

The Ten-Item Personality Inventory—Portuguese version (TIPI-P; Nunes et al., 2018; original version: Gosling et al., 2003) is a brief self-report measure of the Big Five personality dimensions. It consists of 10 items rated on a seven-point Likert scale ranging from 1 (“Strongly disagree”) to 7 (“Strongly agree”). The items are grouped into five dimensions, each assessed by two items: Openness to Experience, Agreeableness, Conscientiousness, Emotional Stability, and Extraversion. In the Portuguese validation study, Cronbach’s alpha coefficients for the five dimensions ranged from 0.39 to 0.72 (Nunes et al., 2018). In the present study, the overall Cronbach’s alpha was 0.53. Given the instrument’s brevity and the use of only two items per dimension, these reliability estimates should be interpreted with caution. Previous research has highlighted psychometric limitations of the TIPI, including low internal consistency and mixed evidence regarding its convergent and structural validity (Thørrisen & Sadeghi, 2023). Accordingly, in the present study, the TIPI-P was used to provide complementary evidence regarding theoretically expected associations between pathological narcissism and broader personality dimensions rather than as a definitive test of convergent validity. The Brief-Pathological Narcissism Inventory—B-PNI (Schoenleber et al., 2015) is a self-report instrument that assesses traits of pathological narcissism through 28 questions with a five-point Likert scale ranging from 0-“Not at all like me” to 5-“Very much like me.” This instrument has two factors: the grandiose narcissism factor (explorativeness, self-sacrifice for self-improvement, and grandiose fantasy), which contains twelve items, and the vulnerable factor (contingent self-esteem, hiding the self, devaluation, right to anger), which contains sixteen items (Schoenleber et al., 2015). The original version of this instrument has a Cronbach’s alpha ranging from α = 0.83 to α = 0.86 for the grandiose narcissism factor and α = 0.93 for the vulnerable narcissism factor, meaning that both factors have adequate internal consistency (Schoenleber et al., 2015). In order to validate this instrument, it was first translated and back-translated, both by a bilingual person, to ensure the structure of the assumption.

### 2.3. Procedures

The study was approved by the Ethics Committee for Research in Social and Human Sciences of the University of Minho (CEICSH), Portugal (protocol code CEICSH 142/2022; approval date: 13 July 2022). After obtaining ethical approval and authorization from the authors of the instruments, the survey was administered online through Google Forms and disseminated via social media and email. The Google Forms questionnaire was configured to limit submission to one response per user, and participants were explicitly informed of this restriction. No additional access-control procedures were implemented. Participants were informed about the study objectives, inclusion criteria, confidentiality, and voluntary nature of participation before providing informed consent. Completion of the survey required approximately 20 min. At the end of the questionnaire, participants were provided with information about psychological support helplines (e.g., SOS Voz Amiga, Conversa Amiga, and Telefone da Amizade).

### 2.4. Data Analysis

Data analyses were conducted using Microsoft Excel and IBM SPSS Statistics version 27 for Windows. Descriptive statistics were initially calculated, and skewness and kurtosis were examined to assess the distribution of the study variables. As the assumptions required for parametric analyses were not met for some variables, non-parametric tests were used where appropriate.

For Objective 1, the factorial structure of the B-PNI was examined through Confirmatory Factor Analysis (CFA), using IBM SPSS AMOS version 27 for Windows. The CFA was conducted at the facet level, with the seven established B-PNI facet scores specified as observed indicators of two correlated latent dimensions: Grandiose Narcissism (Exploitativeness, Self-Sacrificing Self-Enhancement, and Grandiose Fantasy) and Vulnerable Narcissism (Contingent Self-Esteem, Hiding the Self, Devaluing, and Entitlement Rage). This two-factor model was specified a priori, based on the theoretical structure of the B-PNI and previous research examining its organization at the facet level (Schoenleber et al., 2015). The model was estimated using maximum likelihood estimation; no bootstrapping procedure was applied. Model fit was evaluated using the chi-square to degrees of freedom ratio (χ^2^/df), Comparative Fit Index (CFI), Tucker–Lewis Index (TLI), and Root Mean Square Error of Approximation (RMSEA). Following conventional recommendations, χ^2^/df values below 5, CFI and TLI values close to or above 0.90, and RMSEA values below 0.08–0.10 were considered indicative of acceptable model fit (Marôco, 2014). Internal consistency was examined using Cronbach’s alpha coefficients. Construct validity was further examined through associations between the B-PNI dimensions and theoretically related broader personality dimensions and psychopathological symptoms.

For Objective 2, descriptive analyses were conducted to examine the frequency and proportion of participants reporting C-IPV behaviors within the study sample. Overall C-IPV perpetration was operationalized as reporting at least one behavior (score > 0) on any of the three negative Cyberdating Q_A dimensions assessed in the present study: Online Control, Online Jealousy, or Online Intrusive Behaviors. Each dimension was also dichotomized to indicate the absence (0) or presence (1) of at least one reported behavior within that dimension.

For Objective 3, point-biserial correlations were used to examine associations between the dichotomous overall C-IPV perpetration variable and pathological narcissism, considering overall narcissistic traits and the Grandiose and Vulnerable Narcissism dimensions. Correlation coefficients were interpreted considering their magnitude and the substantive context of the associations.

## 3. Results

### 3.1. Confirmatory Factor Analysis of the B-PNI

Prior to the CFA, data were screened for missing values, outliers, and distributional assumptions. No substantial missing data were identified.

Descriptive statistics and correlations among the main study variables are presented in Table 1. Because some variables did not fully satisfy the normality assumptions, Spearman’s rank-order correlations were computed.

For the confirmatory factor analysis (CFA), multivariate normality was assessed and considered adequate (Kurtosis multivariate = 4.38 < 10; Kline, 2005), supporting the use of maximum likelihood estimation.

A confirmatory factor analysis (CFA) was conducted to examine the factorial structure of the Brief Pathological Narcissism Inventory (B-PNI). Preliminary analyses indicated adequate multivariate normality (Kurtosis multivariate = 4.38 < 10; Kline, 2005), supporting the use of maximum likelihood estimation.

The proposed two-factor model demonstrated an acceptable fit to the data. Although the chi-square test was statistically significant, χ^2^(11) = 34.72, *p* < 0.001, additional fit indices supported model adequacy: Comparative Fit Index (CFI) = 0.982, Tucker–Lewis Index (TLI) = 0.965, and Root Mean Square Error of Approximation (RMSEA) = 0.076. These values are consistent with recommended cutoff criteria for acceptable to good model fit (Schreiber et al., 2006).

**Figure 1:**
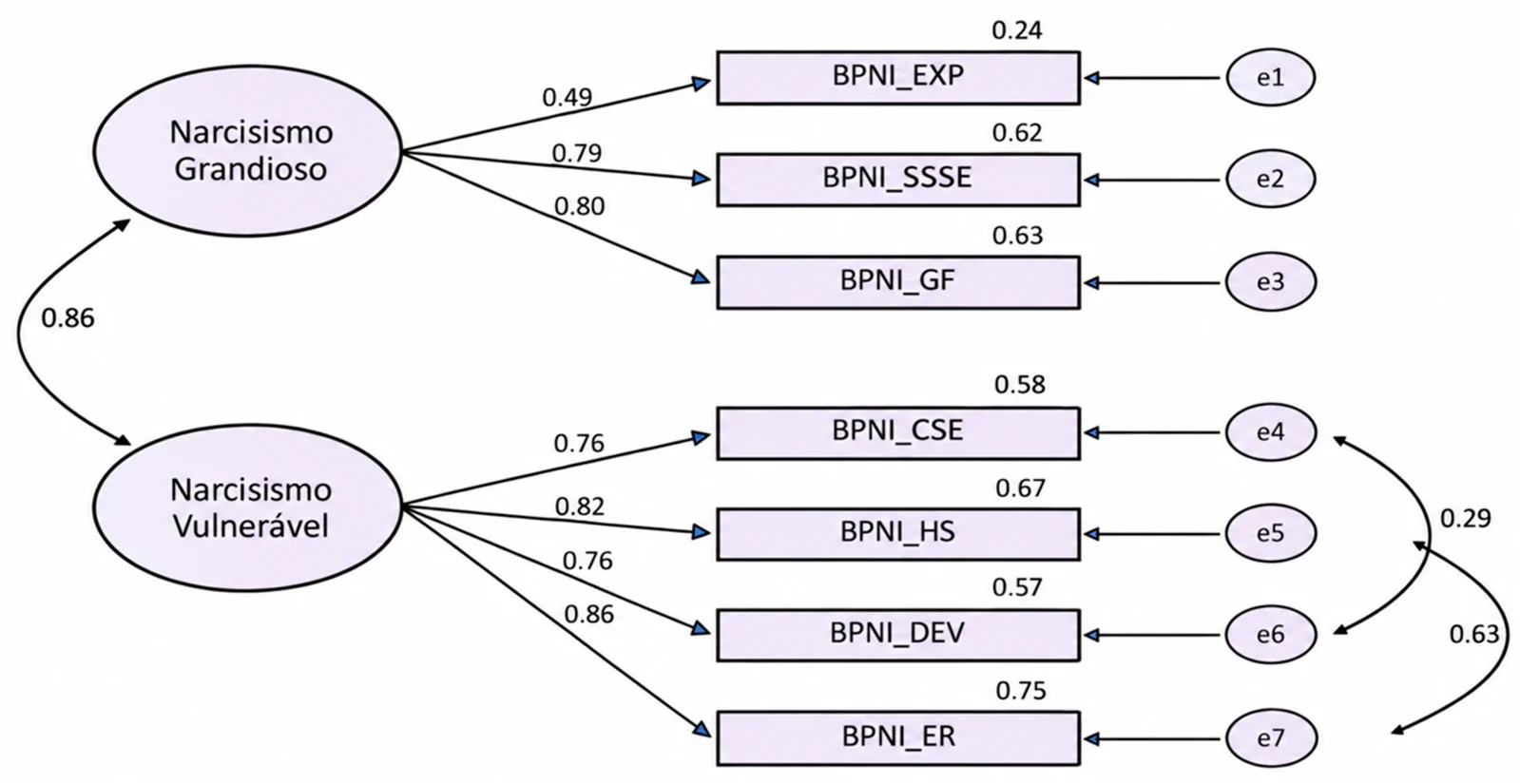
Confirmatory factor analysis of the Brief Pathological Narcissism Inventory (B-PNI), showing standardized factor loadings and correlations between the grandiose and vulnerable narcissism dimensions. Standardized estimates are presented. EXP = Exploitativeness; SSE = Self-Sacrificing Self-Enhancement; GF = Grandiose Fantasy; CSE = Contingent Self-Esteem; HS = Hiding the Self; DEV = Devaluation; ER = Entitlement Rage; e1–e7 = error terms. Curved double-headed arrows represent covariances between the latent factors and between specified error terms. Covariances between error terms were added based on modification indices and theoretical interpretability.

Based on modification indices and theoretical interpretability, a small number of covariances between conceptually related error terms were specified to improve model specification (Marôco, 2014). Figure 1 presents the final CFA model.

The correlation between the two latent factors was high and positive (r = 0.86), indicating substantial shared variance between grandiose and vulnerable narcissism dimensions. Standardized factor loadings were generally adequate and above 0.50, with the exception of the Exploitativeness (EXP) facet, which presented a slightly lower loading (0.49).

### 3.2. Reliability of the B-PNI

Internal consistency analyses were conducted using Cronbach’s alpha coefficients. The grandiose narcissism factor demonstrated acceptable internal consistency (α = 0.72), whereas the vulnerable narcissism factor showed good internal consistency (α = 0.87).

Regarding the grandiose narcissism facets, Cronbach’s alpha coefficients ranged from 0.74 to 0.87, specifically Exploitativeness (EXP), α = 0.74; Self-Sacrificing Self-Enhancement (SSE), α = 0.78; and Grandiose Fantasy (GF), α = 0.87.

For the vulnerable narcissism facets, alpha coefficients ranged from 0.77 to 0.84: Devaluation (DEV), α = 0.77; Contingent Self-Esteem (CSE), α = 0.84; Hiding the Self (HS), α = 0.81; and Entitlement Rage (ER), α = 0.79.

Overall, the B-PNI demonstrated satisfactory internal consistency across both global dimensions and their respective facets.

### 3.3. Construct Validity of the B-PNI

Evidence of construct validity was examined through correlations with the TIPI-P dimensions (see Table 2). Statistically significant, although small, positive correlations were found between grandiose narcissism and all TIPI-P dimensions: Extraversion (rs = 0.136, *p* < 0.01), Openness to Experience (rs = 0.141, *p* < 0.01), Agreeableness (rs = 0.212, *p* < 0.01), Conscientiousness (rs = 0.170, *p* < 0.01) and Emotional Stability (rs = 0.149, *p* < 0.01). Vulnerable narcissism showed a significant positive association only with Agreeableness (rs = 0.166, *p* < 0.01).

Additional evidence regarding the measurement properties of the two B-PNI dimensions was obtained by examining the Average Variance Extracted (AVE) and Composite Reliability (CR). AVE values were satisfactory for vulnerable narcissism (0.64) and reached the recommended threshold for grandiose narcissism (0.50) (Netemeyer et al., 2003). Composite Reliability values were satisfactory for both vulnerable narcissism (0.88) and grandiose narcissism (0.82), exceeding the recommended cutoff value of 0.70 (Netemeyer et al., 2003).

Associations between the B-PNI dimensions and psychological symptomatology were also examined using the Brief Symptom Inventory (BSI; see Table 3). Vulnerable narcissism was positively associated with Somatization (rs = 0.346, *p* < 0.01), Depression (rs = 0.499, *p* < 0.01), and Anxiety (rs = 0.460, *p* < 0.01). Similarly, grandiose narcissism showed positive associations with Somatization (rs = 0.228, *p* < 0.01), Depression (rs = 0.270, *p* < 0.01), and Anxiety (rs = 0.256, *p* < 0.01).

Finally, the correlation between the two latent dimensions obtained in the CFA model was high (r = 0.86), indicating substantial shared variance between grandiose and vulnerable narcissism.

### 3.4. Frequency of C-IPV Perpetration in the Study Sample

Regarding C-IPV perpetration(Table 4), 79.5% (*n* = 295) of participants reported at least one behavior across the three negative Cyberdating Q_A dimensions assessed in the present study. Specifically, 61.5% (*n* = 228) reported at least one online control behavior, 62.0% (*n* = 230) at least one online jealousy behavior, and 31.5% (*n* = 117) at least one online intrusive behavior.

### 3.5. Associations Between C-IPV Perpetration and Pathological Narcissism

Point-biserial correlation analyses revealed positive and statistically significant associations between C-IPV perpetration and pathological narcissism overall (rpb = 0.242, *p* < 0.01), with vulnerable narcissism (rpb = 0.212, *p* < 0.01), and with grandiose narcissism (rpb = 0.238, *p* < 0.01). Although these associations were small in magnitude, the findings indicate that higher levels of pathological narcissism were associated with greater involvement in C-IPV perpetration.

## 4. Discussion

The present study aimed to examine the psychometric properties of the Brief Pathological Narcissism Inventory (B-PNI; Schoenleber et al., 2015) in a Portuguese sample of emerging adults, describe the frequency of self-reported cyber intimate partner violence (C-IPV) perpetration within the study sample, and examine the associations between C-IPV perpetration and pathological narcissism.

The CFA provided support for the a priori two-factor model of the B-PNI examined in the present study, comprising grandiose and vulnerable narcissism. The distinction between grandiose and vulnerable narcissism is consistent with the theoretical structure proposed in the original B-PNI validation study (Schoenleber et al., 2015) and subsequently examined in other cultural contexts (e.g., Malaeb et al., 2023). The fit indices obtained in the present study indicated an acceptable to good model fit, with CFI and TLI values above recommended thresholds and an RMSEA value within the acceptable range (Schreiber et al., 2006). The correlation between the two latent dimensions was high (r = 0.86), indicating substantial shared variance between grandiose and vulnerable narcissism. Although conceptual overlap between these dimensions is consistent with broader models of pathological narcissism (Pincus & Lukowitsky, 2010), the magnitude of this association also reinforces the need for future studies to compare the two-factor model with alternative factorial specifications.

The B-PNI also demonstrated satisfactory reliability. Internal consistency values were acceptable to good for both higher-order dimensions and all facets (DeVellis, 2017), providing preliminary evidence regarding the reliability of the B-PNI among Portuguese emerging adults. Moreover, the AVE and CR indices provided additional evidence regarding the reliability and convergent validity of the two B-PNI dimensions, although the grandiose narcissism dimension reached only the minimum recommended AVE threshold (Netemeyer et al., 2003).

Evidence of construct validity was examined through associations with personality dimensions and psychological symptomatology. Grandiose narcissism showed weak but significant positive associations with all TIPI-P dimensions, whereas vulnerable narcissism was significantly associated only with agreeableness. Similar findings were reported by Zajenkowski and Szymaniak (2021), who observed broader associations between grandiose narcissism and Big Five personality traits, while vulnerable narcissism showed a more restricted pattern of associations. The weak magnitude of the correlations found in the present study should, however, be interpreted cautiously, particularly considering the reduced number of items included in the TIPI-P (Nunes et al., 2018).

Both narcissism dimensions were also positively associated with somatization, depression, and anxiety symptoms. These findings are consistent with the previous literature showing associations between pathological narcissism and psychological distress (Brailovskaia et al., 2020, 2021; Kealy et al., 2018). More specifically, vulnerable narcissism has been associated with anxiety and depressive symptomatology (Brailovskaia et al., 2020), whereas both vulnerable and grandiose narcissism have been found to moderate the association between loneliness and depressive symptoms (Brailovskaia et al., 2021). Similarly, Kealy et al. (2018) demonstrated that pathological narcissism may contribute indirectly to somatic distress.

Regarding C-IPV perpetration, a substantial proportion of participants in the present sample (79.5%) reported at least one behavior across the three negative Cyberdating Q_A dimensions assessed. Online jealousy (62.0%) and online control (61.5%) were the most frequently reported dimensions, followed by online intrusive behaviors (31.5%). These findings are broadly consistent with previous Portuguese studies documenting substantial levels of cyber intimate partner violence among young people and emerging adults (Caridade & Braga, 2019; Caridade et al., 2020; Monteiro et al., 2023), as well as with international research documenting digitally mediated abusive behaviors in intimate relationships (Borrajo et al., 2015; Brown & Hegarty, 2018; Caridade et al., 2019).

The higher frequency of online control and jealousy behaviors relative to online intrusive behaviors may reflect the normalization and social acceptability of monitoring and controlling practices within digitally mediated intimate relationships (Caridade et al., 2020). Indeed, behaviors such as checking a partner’s online activity, monitoring social interactions, or expressing jealousy through digital technologies are often perceived by young people as normative expressions of relational involvement rather than as abusive behaviors. These findings further reinforce the importance of distinguishing between different dimensions of C-IPV when examining patterns of C-IPV perpetration and associated factors.

C-IPV perpetration was positively associated with pathological narcissism overall, consistent with previous studies documenting associations between narcissistic traits and aggressive and controlling behaviors in intimate relationships (Lambe et al., 2018; Pineda et al., 2021). Nevertheless, the magnitude of these associations was weak and should therefore be interpreted cautiously. These effect sizes may reflect genuinely modest associations, consistent with the multifactorial nature of C-IPV, in which personality traits constitute only one component among multiple individual, relational, and contextual influences. This interpretation is consistent with the General Aggression Model (Anderson & Bushman, 2002), which emphasizes that aggressive behavior emerges from the interaction between multiple personal and situational factors.

The results further showed significant associations between C-IPV perpetration and both vulnerable and grandiose narcissism, with associations of similar magnitude. This finding suggests that both dimensions of pathological narcissism may be relevant to understanding C-IPV perpetration, although potentially through different underlying processes. Characteristics associated with grandiose narcissism, such as entitlement, dominance, interpersonal exploitation, and the need for admiration (Miller et al., 2011), may be relevant to controlling and aggressive behaviors enacted through digital technologies. In contrast, the association with vulnerable narcissism may reflect characteristics such as insecurity, rejection sensitivity, and emotional vulnerability, which have been proposed to contribute to controlling and monitoring behaviors in response to perceived threats of abandonment or rejection (Branson & March, 2021; March et al., 2021). These interpretations should nevertheless remain tentative, particularly given the modest magnitude of the observed associations.

More broadly, the literature on narcissism and C-IPV remains inconsistent. Whereas some studies have reported associations primarily with vulnerable narcissism (Branson & March, 2021; March et al., 2021), others have identified associations with grandiose narcissism or with both dimensions (Takezawa et al., 2022; Zerach, 2016). The present findings, showing associations of comparable magnitude for vulnerable and grandiose narcissism, add to evidence suggesting that both dimensions warrant consideration when examining personality-related correlates of C-IPV.

Several limitations should be acknowledged. First, the study relied on a non-probabilistic sample of emerging adults aged 18–25 years, with women substantially overrepresented. These characteristics limit the generalizability of the findings, including the observed frequencies of C-IPV behaviors, which should be interpreted as descriptive of the present sample rather than as population prevalence estimates. The gender imbalance also precluded a robust examination of measurement invariance and gender-specific patterns in C-IPV. Accordingly, future studies should replicate these findings in larger, more gender-balanced samples covering a broader age range of emerging adults and examine measurement invariance across gender and other relevant socio-demographic groups.

Second, some limitations concern the psychometric evaluation and measurement strategy. Although the findings provided support for the a priori two-factor model of the B-PNI examined in this study, only one theoretical model was tested, and the CFA was conducted at the facet level. The findings should therefore not be interpreted as demonstrating the superiority of this structure over alternative factorial solutions. Future validation studies should examine the B-PNI directly at the item level and compare alternative models, including unidimensional and hierarchical specifications. Some Cyberdating Q_A dimensions also showed relatively low internal consistency in the present sample, which may have introduced measurement error and should be considered when interpreting findings involving these dimensions. In addition, although the TIPI-P offered the advantage of reducing participant burden, its brevity entails recognized psychometric limitations, including low internal consistency and mixed evidence regarding convergent and structural validity (Thørrisen & Sadeghi, 2023). Associations with the TIPI-P dimensions should therefore be regarded as complementary evidence of theoretically expected relationships with broader personality traits rather than as a definitive test of convergent validity. Future studies should seek to replicate these findings using more comprehensive measures of the Big Five personality dimensions.

Third, the cross-sectional design precludes causal or directional interpretations, while reliance on self-report measures may have increased susceptibility to response biases. This is particularly relevant for self-reported C-IPV perpetration, given the socially sensitive nature of these behaviors. As no social desirability measure was included, the extent to which responses may have been influenced by social desirability bias could not be assessed. The relatively small associations observed between pathological narcissism and C-IPV should also be interpreted in this context. Although they may reflect genuinely modest associations, consistent with the multifactorial nature of C-IPV, methodological factors may also have attenuated the observed effects. Future research using longitudinal designs, complementary assessment methods, and strategies to assess or minimize social desirability bias would help clarify the magnitude and directionality of these associations.

Finally, the present study did not examine whether the associations between pathological narcissism and C-IPV varied according to gender or relationship characteristics, such as relationship duration or current relationship status. These variables were beyond the a priori analytical objectives of the present study but may contribute to a more nuanced understanding of the conditions under which narcissistic traits are associated with C-IPV. Future studies with sufficiently large and balanced samples should examine these and other potential moderators and consider additional individual and relational factors, such as jealousy, attachment insecurity, emotional regulation, prior victimization, and offline intimate partner violence.

## 5. Conclusions

This study contributes to the literature on pathological narcissism and cyber intimate partner violence by providing preliminary evidence regarding the psychometric properties of the B-PNI in a Portuguese sample of emerging adults. The findings supported the a priori two-factor model examined in the present study and indicated satisfactory reliability and theoretically expected associations with psychopathological symptoms and broader personality dimensions.

The study also documented a substantial frequency of self-reported C-IPV perpetration and controlling behaviors within the study sample, highlighting the relevance of digitally mediated abusive behaviors among emerging adults. Furthermore, the positive, although modest and similar in magnitude, associations between C-IPV perpetration and both vulnerable and grandiose narcissism suggest that narcissistic traits may represent one of several individual factors relevant to understanding cyber intimate abuse.

Overall, the findings suggest that the B-PNI may represent a potentially useful instrument for assessing pathological narcissism among Portuguese emerging adults, although further validation in larger and more diverse samples, including comparisons with alternative factorial models, is warranted.

## Figures and Tables

**Table 1:** Descriptive Statistics for Pathological Narcissism, C-IPV, and Psychopathological Symptoms.

Variable	N	*M*	*SD*
Pathological Narcissism (B-PNI)			
Vulnerable Narcissism	371	27.33	17.97
Grandiose Narcissism	371	28.61	13.00
Total Pathological Narcissism	371	55.94	28.59
Cyber Intimate Partner Violence (C-IPV)			
C-IPV Perpetration	371	0.80	0.40
Online Control	371	0.61	0.49
Online Jealousy	371	0.62	0.49
Online Intrusive Behaviors	371	0.32	0.46
Psychopathological Symptoms (BSI)			
Somatization	371	5.88	6.53
Depression	371	7.89	6.66
Anxiety	371	7.42	6.23
Personality Dimensions (TIPI-P)			
Extraversion	368	8.39	1.99
Openness to Experience	368	5.36	1.52
Agreeableness	368	7.57	1.77
Conscientiousness	368	8.17	2.40
Emotional Stability	368	9.33	2.26

Note. B-PNI = Brief Pathological Narcissism Inventory; C-IPV = Cyber Intimate Partner Violence; BSI = Brief Symptom Inventory; TIPI-P = Ten-Item Personality Inventory—Portuguese Version.

**Table 2:** Spearman Correlations Between the B-PNI Dimensions and TIPI-P Factors.

	E	O	A	C	ES
BPNI Vulnerable	0.098	−0.037	0.166 **	0.087	0.059
BPNI Grandiose	0.136 **	0.141 **	0.212 **	0.170 **	0.149 **

Note. BPNI = Brief Pathological Narcissism Inventory; TIPI-P = Ten-Item Personality Inventory—Portuguese Version; E = Extraversion; O = Openness to Experience; A = Agreeableness; C = Conscientiousness; ES = Emotional Stability. ** *p* < 0.01.

**Table 3:** Spearman Correlations Between the B-PNI Dimensions and BSI Symptoms.

	Somatization	Depression	Anxiety
BPNI Vulnerable	0.346 **	0.499 **	0.460 **
BPNI Grandiose	0.228 **	0.270 **	0.256 **

Note. BPNI = Brief Pathological Narcissism Inventory; BSI = Brief Symptom Inventory. ** *p* < 0.01.

**Table 4:** Frequency of C-IPV Perpetration and Negative Cyberdating Q_A Dimensions.

Variable	*n*	%
C-IPV perpetration	295	79.5
Online control	228	61.5
Online jealousy	230	62.0
Online intrusive behaviors	117	31.5

Note. C-IPV perpetration refers to participants reporting at least one behavior on any of the three negative Cyberdating Q_A dimensions assessed in the present study. Percentages for online control, online jealousy, and online intrusive behaviors represent participants reporting at least one behavior within each respective dimension. These categories are not mutually exclusive, as participants could report behaviors across multiple dimensions; therefore, their percentages are not expected to sum to 100%.

## Data Availability

The data presented in this study are not publicly available due to ethical and privacy restrictions related to the sensitive nature of the data and the protection of participants’ confidentiality. Anonymized data may be made available by the corresponding author upon reasonable request and subject to approval by the relevant ethics committee and institutional regulations.
